# Evaluation of the Pharmaceutical Properties and Value of Astragali Radix

**DOI:** 10.3390/medicines5020046

**Published:** 2018-05-21

**Authors:** Amy G. W. Gong, Ran Duan, Huai Y. Wang, Xiang P. Kong, Tina T. X. Dong, Karl W. K. Tsim, Kelvin Chan

**Affiliations:** 1 Shenzhen Key Laboratory of Edible and Medicinal Bioresources, SRI, The Hong Kong University of Science and Technology, Shenzhen 518057, China; duanran@ust.hk (R.D.); hyw@ust.hk (H.Y.W.); xpkong@ust.hk (X.P.K.); botina@ust.hk (T.T.X.D.); botsim@ust.hk (K.W.K.T.); 2 Division of Life Science, Center for Chinese Medicine, The Hong Kong University of Science and Technology, Clear Water Bay, Hong Kong 100044, China; 3 Department of Pharmaceutical Sciences, Zunyi Medical University, Zhuhai Campus, Zhuhai 519041, China; 4 School of Pharmacy & Biomolecular Sciences, Liverpool John Moores University, Liverpool L3 3 AF, UK; 5 National Institute of Complementary Medicine, Western Sydney University, Sydney, NSW 2560, Australia; 6 Faculty of Science, University of Technology Sydney, Ultimo, NSW 2007, Australia

**Keywords:** traditional Chinese medicine, Astragali Radix, pharmaceutical values

## Abstract

Astragali Radix (AR), a Chinese materia medica (CMM) known as Huangqi, is an important medicine prescribed in herbal composite formulae (Fufang) by Traditional Chinese medicine (TCM) practitioners for thousands of years. According to the literature, AR is suggested for patients suffering from “Qi”- and “Blood”-deficiencies, and its clinical effects are reported to be related to anti-cancer cell proliferation, anti-oxidation, relief of complications in cardiovascular diseases, etc. The underlying cell signaling pathways involved in the regulation of these various diseases are presented here to support the mechanisms of action of AR. There are two botanical sources recorded in China Pharmacopoeia (CP, 2015): *Astragalus membranaceus* (Fisch.) Bge. Var. *mongohlicus*, (Bge.) Hsiao, and *Astragalus membranaceus* (Fisch.) Bge. (Fam. Leguminosae), whose extracts of dried roots are processed via homogenization-assisted negative pressure cavitation extraction. Geographic factors and extraction methods have impacts on the pharmaceutical and chemical profiles of AR. Therefore, the levels of the major bioactive constituents of AR, including polysaccharides, saponins, and flavonoids, may not be consistent in different batches of extract, and the pharmaceutical efficacy of these bioactive ingredients may vary depending on the source. Therefore, the present review mainly focuses on the consistency of the available sources of AR and extracts and on the investigation of the biological functions and mechanisms of action of AR and of its major bioactive constituents. Furthermore, it will also include a discussion of the most popular AR composite formulae to further elucidate their chemical and biological profiles and understand the pharmaceutical value of AR.

## 1. Introduction

Huangqi (Astragali Radix, AR) is one of the most popular herbal medicines in traditional Chinese medicine (TCM), firstly recorded in “Shennong Bencao Jing” (translated as “The Devine Farmer’s Materia Medica”, which is the Classic of Herbal Medicine in ancient TCM practice). AR tastes sweet and tepid and is extensively used to tonify spleen and lung functions according to the TCM treatment principles. TCM considers that AR can enrich “Qi” (vital energy), treat stagnant blood flow due to qi deficiency, and improve “Yin” deficiency by promoting diuresis to remove oedema due to inadequate transformation of dampness and “Qi”. In the modern pharmaceutical principle, AR enhances the immune system and the blood circulation. Details of these properties and their mechanisms of action will be reviewed and summarized in the following paragraphs.

**Figure 1:**
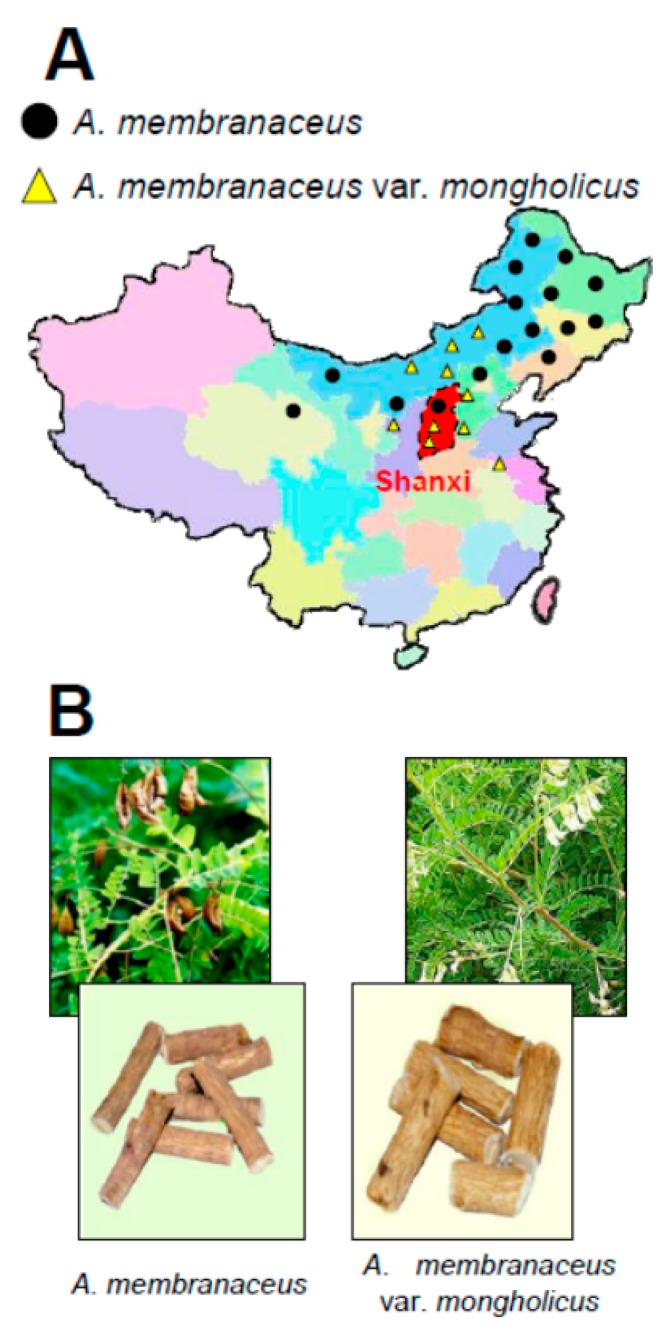
Sources of Astragali Radix (AR) and plant morphology. (**A**) AR produce source is widely present in the mainland of China. *Astragalus membranaceus* is mainly produced in the northwest and west of China (black dots). *A. membranaceus* var. *mongholicus* is greatly produced in the west of China, (yellow triangles). AR collected from Shanxi, China, highlighted in red, is believed to be “Di Dao” material. (**B**) Authentic plants of *A. membranaceus* (left) and *A. membranaceus* var. *mongholicus* (right).

The genus *Astragalus* L. consists of 278 species, 2 subspecies, 35 varieties, and 2 forma found predominantly within China [1,2]. Two authentic botanical sources of AR recorded in Chinese Pharmacopoeia are *Astragalus membranaceus* (Fischer) Bunge and *A. membranaceus* (Fisch.) Bunge var. *mongholicus* (Bunge) P. K. Hsiao (CP, 2015). *A. membranaceus* and *A. membranaceus* var. *mongholicus* are mainly cultured in Inner Mongolia, Shanxi, Gansu, and Heilongjiang provinces in the north and the northeast parts of mainland China (Figure 1) [2,3]. In fact, *A. membranaceus* and *A. membranaceus* var. *mongholicus* share a basic morphology (Figure 1). Furthermore, *A. membranaceus* and *A. membranaceus* var. *mongholicus* share close similarities of chemical components, i.e., isoflavonoids, saponins, polysaccharides, γ-aminobutyric acid (GABA), and various trace elements, and of genetic components, represented by the internal transcribed spacer sequences of nuclear ribosomal DNA [2,4,5]. Indeed, random amplified polymorphic DNA primers were employed to reveal DNA fingerprinting differences between *A. membranaceus* and *A. membranaceus* var. *mongholicus*. The primer pairs HG3 and HG4 are specific for *A. membranaceus*, whereas HG7 and HG8 can identify *A. membranaceus* var. *mongholicus* [6]. Furthermore, microscopic characteristics, such as layers of phellem, continuing lignified xylem bundles within spring wood, and lignified parenchyma cells in the central part of the xylem, are other major tools to differentiate these two species from others [7]. Besides, annual rings can be identified in the roots of both species and provide critical information for determining the age of a sample [8]. Therefore, microscope analysis could be a key technique to examine the differences between *A. membranaceus* and *A. membranaceus* var. *mongholicus*.

## 2. Chemical Determination of Different Plant Parts of Astragali Radix

Historically, the root, but not other plant parts, of *A. membranaceus* has pharmaceutical properties. Kim and co-workers compared the chemical and genetic composition of aerial parts, including flower and stems, and root parts of *A. membranaceus*. A total of 10 mevalonate pathways involved in astragaloside biosynthesis were found and identified by Illumina/Solexa HiSeq2000 [9]. The accumulation rates of astragalosides were different in various plant organs. Most genes were highly expressed in the root rather than in the stem and leaf [9]. In particular, the concentration of astragaloside IV was distributed in the following order: root (0.58 mg/g) > flower (0.27 mg/g) > stem (0.23 mg/g) > leaf (0.04 mg/g) [10]. In line with this report, the content of calycosin-7-*O*-β-d-glucoside content was enriched as follows: root (4.88 μg/g) > stem (3.86 μg/g) > leaf (2.0 μg/g) > flower (not detected) [9]. These chemical results support fully the pharmaceutical values of the root of *A. membranaceus.*

In fact, the roots of *A. membranaceus* are divided into three major classes: seedling roots, adventitious roots, and hairy roots. Their study revealed that the total content of astragalosides found in the adventitious root was the lowest, whereas the seedling root had the highest content [11]. However, calycosin, one major bioactive isoflavonoid, and calycosin-7-*O*-β-d-glucoside accumulated the most in the seedling root [11]. Interestingly, the total concentration of astragaloside in the periderm was about eight-fold higher in the cortex and about 30-fold higher than in the xylem [12]. The dry weight percentages of total saponins in primary roots were ~40% in the periderm, ~50% in the cortex, and 9.30% in the xylem, respectively [12].

## 3. The Optimization of Extraction of Astragali Radix

In order to search the best extraction method for AR, the roles of background electrolyte concentrations, organic solvents, pH, surfactant concentrations, temperature, and voltage on the separation procedure were systematically identified and compared [8]. The optimized extract condition was found to be the micellar phase containing 100 mM sodium cholate, 25% (*v*/*v*) acetonitrile, and 20 mM H_3_BO_3_ buffer at pH 9.2. Furthermore, repeatability parameters, i.e., intra-day and inter-day precisions, were determined and resulted to be below 4.42% [8]. In 2014, Kim et al. reported that the eight constituents isolated from an 80% methanol AR extract showed a better inhibition of matrix metalloproteinases (MMP) production in IL-1-induced osteoarthritis models, than a water extract of AR [13].

The novel strategy involving pressurized liquid extraction (PLE), microwave-assisted acidic hydrolysis (MAAH), and comprehensive chromatography served as an effective method to increase the polysaccharide extraction yield. The quantification of twelve saccharides, i.e., glucose, free sucrose, fructose, etc. was performed by gas chromatography–mass spectrometry (GC–MS) and HPLC. The results showed that *A. membranaceus* dried powder contained about 108.5 mg/g free sucrose and lesser amounts of glucose (9.6–26.0 mg/g) and fructose (8.7–22.9 mg/g). Hence, this extraction method was much more efficient than the traditional extraction method [14]. Homogenization-assisted negative pressure cavitation extraction (HNPCE) is another effective and innovative method to extract polysaccharides from AR. The optimal extraction parameters were determined as: homogenization time 70 s, negative pressure −0.068 MPa, extraction time 53 min at 64.8 °C, which increased the polysaccharide yield around 15% [15]. Yin et al. (2012a) have established a green and interesting method based on an aqueous diphase solvent system, consisting of PEG1000-MgSO_4_-H_2_O, which purified polysaccharides by using 76.5% of galacturonic acid, 7.7% of galactose, 4.2% of arabinose, etc. [16]. Therefore, we suggest to employ HNPCE to generate the polysaccharide-enriched AR fractions.

## 4. The Pharmaceutical Value of AR Extract and AR Major Ingredients

AR is one of the most popular herbal medicines used worldwide, possessing tonic, hepatoprotective, diuretic, and expectorant properties, according to the Chinese Pharmacopoeia (CP Volume 1 of 2015 Edition) [2]. It has been shown to have anti-oxidation properties, regulate the immune function, mitigate cardiovascular diseases, inhibit liver fibrosis, and other pharmaceutical functions [17]. 

### 4.1. The Anti-Oxidative Actions of Astragali Radix and Its Major Constituents

Oxidative stress plays a key role in the pathogenesis of various diseases, and anti-oxidants compounds could protect cells and tissues from oxidative stress by removing reactive oxygen species [18]. The anti-oxidative functions of AR and of its pharmaceutical constituents are summarized in Table 1. In 1999, Toda & Shirataki indicated that the anti-oxidative functions of AR were superior or similar to those of the positive controls butyl hydroxytoluene and α-tocopherol [19]. Furthermore, AR was reported to adapt to water stress during the growth season by enhancing the activity of anti-oxidant enzymes and accumulating osmotic agents [20]. The AR extract could mitigate the telomere shortening rate of lung diploid fibroblasts 2BS, which is hypothesized to be related to a reduction of DNA damage and the improvement of DNA repair ability [21]. Moreover, the declined telomere shortening rate, the diminished DNA damage, as well as the upregulated DNA repair ability triggered by AR extract are believed to be responsible for the delay of replicative senescence [21]. Animal studies have shown, by histopathology and immunohistochemistry, upregulated lipid peroxidation levels in the cerebral cortex, hippocampus regions, and injured brain tissue in a whisker rat model of removal-induced psycho-emotional stress [22]. However, the oral administration of AR extract could significantly decrease the content of these biomarkers [22]. The activation of NF-κB was predominant in the untreated group, while it was significantly suppressed by the application of an AR water extract in cerebral cortex and hippocampus regions [22].

The anti-oxidant activities of three new flavonoids, including one aurone and two chalcones, were investigated in 2,2-diphenyl-1-(2,4,6-trinitrophenyl) hydrazyl free radical scavenging assays [23]. The IC_50_ of two chalcones were 35.9  ±  1.1 and 12.2  ±  1.1 µM, respectively [23]. The anti-oxidative functions of formononetin and ononin were much stronger than those of tert-Butylhydroquinon (TBHQ) or butylated hydroxyanisole (BHA) when determined by 2,2-Diphenyl-1-Picrylhydrazyl (DPPH), 2,2′-azino-bis(3-ethylbenzothiazoline-6-sulphonic acid (ABTS), ferric reducing ability of plasma (FRAP), and lipid peroxidation inhibition assays [24,25,26]. Indeed, the combination of formononetin, ononin, calycosin, and calycosin-7-*O*-β-d-glucoside also exhibited anti-oxidative therapeutic effects in anemic rats, as indicated by the serum levels of SOD and GSH-Px [27].

Astragalosides also showed pharmaceutical value as anti-oxidants (Table 1) and could decrease the level of high-mobility group box 1 protein, a highly expressed protein that regulates acute inflammation in mouse models [28]. On the other hand, astragalosides prevented renal and mitochondrial dysfunctions through their anti-oxidative effects in crush syndrome rat models [29]. This modulatory function was hypothesized to be related to the TLR4/NF-κB pathway [30]. In addition, astragalosides were shown to improve pulmonary inflammatory diseases by interacting with autophagy and PERK-eIF2α pathways [31].

The polysaccharide-enriched AR fractions showed promising therapeutic effects as anti-oxidants both in vivo and in vitro [32,33,34]. In hyperlipidemia and oxidative stress-induced rat models, the serum levels of SOD and GSH-Px activity declined after oral administration of a polysaccharide-enriched AR fraction for four weeks [33]. In vitro studies have shown that AR polysaccharides decreased SOD, GSH-Px, and catalase activities in cultured H_2_O_2_-induced MRC-5 cells mimicking oxidative damage models [34]. Another research group reported that a polysaccharide-enriched constituent also played a part in the therapeutic effects on oxidative damage in the skeletal muscles of rats after exhaustive exercise [35]. The rats were randomly divided into four groups. Group 1 was the control group, and groups 2 to 4 received different doses of AR polysaccharides for 30 successive days. Skeletal muscle samples were collected to analyze enzymatic activities. The results indicated that the polysaccharide-treated groups had significantly decreased MDA, SOD, GSH-Px, and CAT contents compared to the negative control [35]. The in vivo anti-aging functions of the polysaccharides were determined by Li et al. [36]. The back of the neck of mice was injected with D-Gal for seven weeks to mimic aging. After polysaccharide treatment at the dose of 200 and 300 mg/kg/day, the serum levels of SOD, CAT, GSH-Px, and anti-hydroxyl radical activity were dramatically upregulated in a dose-dependent manner [32].

### 4.2. The Immune Functions of Astragali Radix and Its Biological Ingredients

AR extracts have shown considerable immunomodulatory properties both in vitro and in vivo [37] (Table 2). After oral and intracolonic treatments with AR extracts, a decrease in colonic lesion areas and histological damage score and an amelioration of the colonic myeloperoxidase activity were detected in Sprague–Dawley rats [38,39]. Western blot data demonstrated that AR was capable of diminishing the levels of some cytokines, i.e., TNF-α and IL-1β, and of other immune-specific regulators, such as. P-selectin and ICAM-1 [38,39]. Furthermore, AR was also reported to have non-specific immune effects on tilapia [40]. The plasma levels of extracellular superoxide anion production and phagocytosis were examined after feeding three-month-old tilapia with an AR extract. The results showed that the AR extract was capable of modulating the innate immune system by upregulating lysozyme activity instead of altering the respiratory burst activity [40]. Clinically, leukocyte, platelet, and cytokine responses as well as body temperature and blood pressure were examined in healthy individuals after consuming an extract of *A. membranaceus* [41]: monocytes, neutrophils, and lymphocytes counts were increased in a dose-dependent manner after 8–12 h from the extract administration. Dynamic changes in the concentration of circulating cytokines, i.e., interferon-γ, TNF-α, IL-13, IL-6, and soluble IL-2R, were observed [41].

An aqueous extract of AR could trigger apoptosis of H22 tumor cells by enhancing Bax/Bcl-2 ratios, which was confirmed both by immunoblotting and flow cytometry [42]. Inhibition of tumor cell proliferation was detected in various cell lines. The inhibition rates of AR extracts in AGS, KATOIII, HT29, MDA231, MEL7, and MEL14 cells were 68.25%, 62.36%, 22.8%, 27.69%, 2.85%, and 5.14%, respectively [43]. AR suppressed DNA synthesis by 87.33% at the concentration of 50 μg/mL in AGS cell line. The effects of a co-treatment of AR with conventional cancer therapy were determined on the 798 breast cancer patients. AR combined with conventional therapy is believed to be efficacious in improving the quality of life in late-stage breast cancer patients and in decreasing the number of hot flashes [44].

After oral administration of AR polysaccharides in cyclophosphamide-induced rat, spleen weight, peripheral white blood cell counts, and T cell and B cell proliferation responses were significantly upregulated [45]. Furthermore, splenic nature killer cell activity and peritoneal macrophage phagocytosis were also dramatically increased [45]. In humans, the intake of AR extract promoted immunomodulating effects by upregulating the proliferation of peripheral blood mononuclear cells, as well as inducing interleukin production [46]. Dose-dependent stimulations of IL-10, IL-12, and IL-2 were markedly observed after application of AR polysaccharides, compared to a negative control [46]. The bioactive constituents of AR also showed the ability to induce TNF, GM-CSF, and NO productions in cultured macrophages via the activation of the NF-κB pathway [47]. Moreover, AR-enriched polysaccharides could increase the sensitivity to cisplatin (DDP) and vinorelbine in lung cancer patient, prolong their life span, and increase their life quality by mitigating the toxicity of the chemotherapeutic drugs [48]. In a clinical trial, 136 patients suffering from non-small-cell lung cancer (NSCLC) were enrolled for the duration of two years [48]. Patients were randomized into two groups: an anti-cancer drug group (DDP and vinorelbine) and an anti-cancer drug–AR polysaccharide co-treatment group. After two years of treatment, several indexes showed significant differences, i.e., physical function (*p* = 0.01), nausea and vomiting (*p* < 0.001), fatigue (*p* < 0.001), pain (*p* = 0.007), and loss of appetite (*p* = 0.023) [48]. In nude mice, the AR-generated polysaccharides were capable of decreasing tumor sizes and increase the expressions of the apoptosis markers cleaved-caspase 3/9 [49]. Increased IgM antibody production in aged mice receiving AR polysaccharides was also confirmed [50]. In aged mice, i.e., 36- and 60-week-old mice, the antibody levels were significantly decreased to about 70% and 60%, respectively, compared to those of 10-week-old mice. IgM production was significantly upregulated in 36- and 60-week-old mice after treated with AR [50]. The immune function enhancing properties of AR polysaccharides could be interpreted. One the other hand, the role of AR-derived polysaccharides in auto-immune diseases has been explored. Chronic inflammation is believed to be the major component of autoimmune diseases development [51,52]. In vitro data revealed that the minimum anti-inflammatory concentration of polysaccharides was 100 μg/mL in LPS-induced Caco-2 cells [53]. The anti-inflammatory effect was revealed in cultured LPS-inducted macrophages by detecting NO and the protein expression levels of IL-1β, IL-6, and TNF-α [54,55]. A polysaccharide-enriched AR fraction suppressed IL-1β level by ∼20%, IL-6 expression by ∼15%, and TNF-α level by ∼25% in LPS-induced macrophages, respectively [55]. Reduced cell accumulation, swelling, joints arthritic index, and serum concentrations of TNF-α and IL1-β were observed in arthritis rat models [56].

The effects of flavonoids derived from AR on the immune functions were determined in Raw 264.7 cells [57]. Four newly isolated compounds, i.e., (−)-methylinissolin 3-*O*-β-d-(6′-acetyl)-glucoside, (−)-methylinissolin 3-*O*-β-d-{6′-[(*E*)-but-2-enoyl]}-glucoside, and calycosin 7-*O*-β-d-(6″-acetyl)-glucoside and (−)-methylinissolin 3-*O*-β-d-glucoside, showed inhibitory effects on NO production in Raw 264.7 cells after LPS-induced chronic inflammation [57]. The inhibitory effects of the newly identified compounds isoliquiritigenin and liquiritigenin on LPS-stimulated bone marrow-derived dendritic cells were investigated [58]. These two compounds exhibited inhibitory effects on LPS-induced IL-6 and IL-12 productions, with IC_50_ values ranging from 2.7 μM to 6.1 μM [58]. Isoliquiritigenin also showed a moderate suppression function on LPS-stimulated TNF-α production, with an IC_50_ value of 20.1 μM. Furthermore, the AR flavonoids calycosin and calycosin-7-*O*-β-d-glucoside accelerated glomerular endothelial cell apoptosis rate [59]. Immunomodulatory functions of AR flavonoids were also well reported in vivo [60]. AR flavonoids could promote lymphocyte proliferation, increase T cell number, modulate T cell subsets disorders, and elevate LAK activity induced by IL-2 in immunosuppressed mice [60,61,62,63].

### 4.3. Protective Effects on Cardiovascular Diseases

The AR herbal extract dramatically decreased total cholesterol and LDL cholesterol and aortic fatty streak area levels; on the other hand, it exhibited the potential ability of increasing HDL cholesterol levels in atherosclerotic rabbits [64]. Besides, the therapeutic functions of AR on atherosclerosis in the aortic endothelium were determined on apolipoprotein E-deficient (apoE−/−) mice [65]. Upregulated expression of VCAM-1 and phosphorylation of NF-κB were the hallmarks of AR, as indicated in the model group mice: Immunofluorescence analysis confirmed the reduced expression of the adhesion molecules and the expression of macrophages in the aortic endothelium in AR-treated apoE−/− mice [65]. In vitro data showed that this ancient herbal medicine could scavenge superoxide and hydroxyl radicals in a concentration- and time-dependent manner [66]. Furthermore, it could effectively suppress free radical formation in ischemia-reperfusion models [64].

AR showed promising effects in improving biochemical and histological changes of heart failure [66]. The therapeutic activities were categorized as: suppressing lipid accumulation via adenosine monophosphate-activated protein kinase activation, increasing LDL receptor expression, alleviating lipid peroxidation, and decreasing inflammatory cytokines production levels [66]. Clinical data indicated that circulating endothelial cells (CEC) and production of endothelin-1 (ET-1) and malondialdehyde (MDA) in the internal jugular vein of Binswanger’s disease patients were dramatically increased after AR treatment, compared to the negative control [67]. Nevertheless, serum NO concentration significantly declined in the AR group [67]. AR is believed to be effective in protecting vascular endothelial cells in Binswanger’s disease patients.

### 4.4. Therapeutic Effects of Astragali Radix on Liver Fibrosis

Intraperitoneal injection of 50% CCl4 twice a week for two months and intraperitoneal injection of dimethylnitrosamine (DMN) are the typical methods for inducing liver fibrosis in Sprague–Dawley rats [68,69]. The hallmarks of liver cirrhosis include high concentrations of aspartate aminotransferase (AST), alanine aminotransferase (ALT), hexadecenoic acid (HA), laminin (LN), procollagen type III (PCIII), hydroxyproline (Hyp), GSH-Px, MDA, SOD, and transforming growth factor β1 (TGF-β1) in the serum. AR could significantly reduece the high levels of these biomarkers in the serum [69]. Hematoxylin–eosin and Masson’s trichrome staining, the classical staining methods for monitoring histopathological changes, confirmed that after administration of AR, the damage to the liver function was decreased [69]. Studies have shown that AR was able to suppress TGF-β1, α-SMA, collagen I, and collagen III expression, block the phosphorylation of Smad2/3, and enhance the expression Smad7, the specific inhibitor of TGF-β1 [69].

### 4.5. The Erythropoietic Functions of Astragali Radix and Its Major Constituents

The angiogenic function of the polysaccharide-enriched AR fraction was revealed in zebrafish. A cocktail containing 300 nM VEGFR tyrosine kinase inhibitor II was applied to Tg(fli-1a: EGFP)y1 and Tg(fli-1a:nEGFP)y7 embryos for 3 h, to induce the loss of blood vessels. After challenging with a polysaccharide-enriched AR fraction, a rescue effect was shown by a statistically significant increase of blood vessels in a dose-dependent manner [70].

Furthermore, our group has investigated the erythropoietic functions of the four major flavonoid constituents of AR (formononetin, ononin, calycosin, and calycosin-7-*O*-β-d-glucoside) by monitoring the expression of erythropoietin (EPO) and its upstream regulator, hypoxia-induced factor (HIF-1α) in cultured HEK293T cells. These four flavonoids could upregulate EPO and HIF-1α at both the transcriptional and the translational levels [71], as shown in the cyclophosphamide-induced anemic rats after treatment with the combined flavonoids; enhanced content of red blood cells, white blood cells, and hemoglobin, and increased hematocrit were significantly observed [27].

### 4.6. Other Pharmaceutical Properties of AR and Its Ingredients

The anti-obesity function of a polysaccharide-enriched AR decoction has been also revealed both in vivo and in vitro [72,73]. Decrease in body weight, improvement in insulin sensitivity, and a mitigation of fatty liver were recorded after polysaccharide administration to type 2 diabetes rats [72]. Agyemang and co-workers observed that AR polysaccharides suppressed the crucial regulators of endoplasmic reticulum stress, such as PERK, ATF-6, and XBP1 in type 2 diabetes rat [73]. Other groups revealed that the AR polysaccharides had a negative role in modulating the GLUT4/PKB glucose transportation pathway in insulin-resistant KKAy mice [74].

The anti-cancer function of AR saponins has been demonstrated, as shown in Table 3. AR saponins inhibited cancer cell proliferation both in cell and in animal models. In HT29 colon adenocarcinoma cells, treatment with AR saponins upregulated cleaved PARP, caspase 8, and NAG-1 levels [75,76]. A total AR saponin fraction triggered PTEN expression and downregulated mTOR expression via blocking NFκB and DNA binding activity [76]; it decreased the levels of VEGF and bFGF in a time- and dose-dependent manner [77]. In vivo studies were performed in colon cancer cell HT29-xenografted nude mice [75,77]. The tumor regression rate was about 35% after treatment with AR saponins, without alterations of mice body weight [75]. The translational levels of p-Akt, p-mTOR, VEGF, VEGFR1, and VEGFR2 were decreased in experimental rat tumor tissues [77]. The co-treatment of astragaloside IV with indoleamine 2,3-dioxygenase (IDO), a tryptophan-catabolizing enzyme triggering immune tolerance, could enhance the immune response by suppressing regulatory T cells and enhancing cytotoxic T lymphocytes; therefore, astragaloside IV might be effective in blocking cancer cell proliferations [78,79].

Astragaloside II was capable of inducing bone matrix formation and remodeling, as shown in Table 3. Astragaloside II exhibited osteogenic functions throughout the whole osteoblast differentiation process [80]. In bone development, astragaloside II induced the expression of master bone matrix regulators, such as BMP-2, Smad1/5/8, ERK1/2 and p38, and Cbfa1/Runx2 [80].

The pain reliving pharmaceutical value of AR was also widely reported. Administration of AR extracts significantly suppressed oxaliplatin-trigged hypersensitivity and promoted rescue mechanisms, preventing damages of the nervous tissue and the triggering of chronic pain [81]. Oxaliplatin enhanced the superoxide anion production both in the stable cell line SH-SY5Y and in primary cortical astrocytes. Administration of AR extracts showed protective effects against oxaliplatin-induced lipid peroxidation, carbonylated proteins, and DNA oxidation [82]. Furthermore, a single administration of a hydroalcoholic AR extract dramatically suppressed both sodium mono-iodoacetate- and complete Freund’s adjuvant-induced pain, with over 70% and 90% of pain relief, in rat models with articular damage resembling osteoarthritis and rheumatoid arthritis, respectively [83].

## 5. Discussion

This review has pointed out the importance of obtaining reliable starting materials for herbal extracts of AR, which are suitable for screening its major bioactive chemicals. It has also summarized the pharmaceutical properties of the CMM, its concentrated extracts, and their individual chemical constituents and purified polysaccharides. These pharmaceutical activities may be useful for the further development of good quality herbal medicines useful in areas where conventional pharmaceutical medicines experience difficulties in controlling chronic diseases, or as nutraceuticals for good health. However, a single CMM is less efficient than well-used composite formulae consisting of several CMMs in combination, because of the multi-targeting properties and synergic or complementing effects of the combined ingredients.

In the clinical practice of TCM, AR is often combined with other CMMs in composite herbal formulae (Fufang) for the treatment of various diseases, according to patients’ individual constitution and symptoms. Indeed, its roles in the different prescription formula are not the same. The principles for composing a TCM prescription, as first described in the Huang Di Nei Jing (The Inner Canon of the Yellow Emperor), stipulate that the prescription may include four different CMMs. They are the *Emperor or Principal* (*Jun* CMM*),* the *Adjuvant* (*Chen* CMM), the *Assistant* (*Zuo* CMM), and the *Guide* (*Shi* CMM), according to the different roles they play in the prescription. At times, some CMM may have two or more roles depending on their diverging properties. The four CMMs, supplementing one another, exert a curative role together. However, not every prescription is composed of four kinds of CMMs; there may be less or more than four, with the *Emperor* CMM being the dominant one, depending on the diseases, the characteristics of the CMMs, and the therapeutic needs. AR is often used as the *Emperor* CMM with little or no toxicity in most TCM formulae. The following three examples illustrate the works our team has researched in detail.

Yu Ping Feng San (YPFS), an ancient TCM prescription, consists of AR, Atractyldis Macrocephalae Rhizoma, and Saposhnikoviae Radix; here, AR plays the major functions in YUPS by enriching “*Yang Qi*” of spleen and lungs and tonifying “*Wei Qi*” *of* the stomach [84,85]. Our research team observed that YPFS was capable of modulating the immune system and it exhibited the possibility of enhancing the innate immune system against bacterial and viral invasions [84]. The co-treatment of YPFS and Cisplatin (DDP) showed potential efficacy in reducing DDP-resistance in NSCLC cells by increasing the intracellular DDP content [85]. In vivo data showed that the co-treatment of DDP and YPFS reduced tumor size by more than 80% in tumor-bearing mice, compared to DDP alone [85].

Buyang Huanwu prescription (BYHWD) is another popular ancient formula consisting of seven CMMs (AR, Angelica Sinensis Radix [ASR], Chuanxiong Radix, Paeoniae Rubra Radix, Persicae Semen, Carthami Flos, and Pheretima) which is designed to treat complications after cerebrovascular accident, paralysis, stroke, and their related complications. In this formula, AR also plays the *Emperor* function. The administration of BYHWD in chronic denervation models significantly increased the axonal regenerative abilities and neurite outgrowth [86]. This formula could also reduce cerebral ischemia/reperfusion damages in animal experiments [87,88,89].

Danggui Buxue Tang (DBT), consisting of two CMMs (AR and ASR), is a widely prescribed classical and simple formula. The pharmaceutical value of this prescription has been reported widely [90]. AR acts as the *Emperor* role. Previous studies have confirmed that DBT is able to improve the cardiovascular function by stimulating NO production in endothelia cells [54]. Immune enhancing functions of DBT were detected both in vitro and in vivo, as observed in cultured T lymphocytes; after application of DBT, cell proliferation was markedly induced, with the release of IL-2, -6, and -10, and the phosphorylation of ERK [60]. On the other hand, DBT could not only withstand significantly the reduction of blood cells by immune mediation, but also stimulate the growth of bone marrow cells and increase the weight of haemopoietic progenitors in the bone marrow in rats [91]. Furthermore, DBT treatment showed a greater improvement of clinical symptoms, such as decreased skin thickness and scratching behavior in a rat model and decline of the total serum IgE levels and mast cells counts, when compared to the control group and to the single-AR-extract-treated group. The levels of cytokines and inflammatory mediators were significantly decreased in the DBT groups [92]. In vivo monitoring of the biomarkers together with in vitro investigations of cell expression/signaling responses are required to elucidate the biological complexity of the composite herbal formula.

The research on composite formulae is only possible with the advances in biomedical, chemical, and computational technology, by employing multidisciplinary approaches for investigating evidence-based aspects of TCM practice. Research linking a specified starting CMM, the relatively new systems biology, and experience-based TCM principles is vital to elucidate the complexity of TCM prescriptions [93]. More research is needed to probe the role of each chemical present in AR and its composite prescriptions, using systems approaches. This will be the future direction for the advancement of TCM research.

## 6. Conclusions

The chemical compositions of various plant parts of *A. membranaceus* and *A. membranaceus* var. *mongholicus* were compared. Homogenization-assisted negative pressure cavitation extraction is considered the optimal extraction method for polysaccharide-enriched AR to obtain consistent chemical ingredients for biological investigations. The pharmaceutical values of AR were summarized. The key values include immunomodulation, anti-oxidation activation, cardiovascular protection, inhibition of liver fibrosis, stimulation of blood regeneration, anti-obesity action, and pain-relieving properties. We believe that AR is one the most important natural medicines of TCM herbal composite formulae, and its promising biological functions should be further explored and tested for clinical application in conditions where conventional medicines may not be efficacious.

## Figures and Tables

**Table 1:** The anti-oxidative functions of AR and its major constituents.

Working Parts	Biological Functions	Model	References
**Astragaloside**	Against oxidation of linoleic acid	In vitro	[19]
	Enhancing anti-oxidant enzymes activities and accumulating osmotic agents	In vitro	[20]
	Improving DNA repair abilities	In vitro	[21]
	Upregulating lipideroxidation levels	In vivo	[22]
**Flavonoids**	Enhancing free radical scavenging activities	In vitro	[23]
	Stimulating lipid peroxidation inhibition levels	In vitro	[24,25,26]
	Decreasing SOD and GSH-Px contents	In vivo	[27]
**Saponins**	Declining high-mobility group box 1 protein content	In vivo	[28]
	Preventing renal and mitochondrial oxidative-induced dysfunctions, possibly through the TLR4/NF-κB pathway	In vivo; In vivo	[29,30]
**Polysaccharides**	Decline of SOD and GSH-Px levels	In vivo	[33,35,36]
	Decrease of SOD, GSH-Px, and catalase activities	In vitro	[34]

SOD: Super oxidase dimutase; GSH-Px: Selenium dependent glutathione-peroxidase.

**Table 2:** The immunomodulatory functions of AR and its major constituents.

Working Parts	Biological Functions	Model	References
**Astragalosides**	Decrease colonic lesion area and histological damage	In vivo	[38,39]
	Enhance non-specific immune response	In vivo	[40]
	Increase of monocytes, neutrophils, and lymphocytes counts	In vivo	[41]
	Increase Bax/Bcl-2 ratio	In vitro	[42]
	Suppress proliferation of various cancer cell types	In vitro	[43]
	Enhance breast cancer patients’ life span and increase their life quality	In vivo	[44]
**Polysaccharides**	T cell and B cell proliferation	In vivo	[45]
	Cytokine upregulation	In vitro	[46]
	Regulation of GM-CSF and NO productions and modulation of Akt pathway	In vitro	[47]
	Prolong cancer patient’s lifespan	In vivo	[48]
	Stimulate tumor cell apoptosis	In vitro	[49]
	Enhance IgM antibody production	In vivo	[50]
	Suppress chronic inflammation cytokine level	In vitro	[56,57,58,59,60]
	Increase synovial cell apoptosis rate	In vivo	[61]
**Flavonoids**	Suppress NO and chronic inflammatory mediator release	In vitro	[62]
	Inhibit LPS-stimulated cytokine production in bone marrow-derived dendritic cells	In vitro	[58]
	Accelerate cancer apoptosis rate	In vitro	[59]
	Prolong cancer patient’s lifespan	In vivo	[48]
	Stimulate lymphocyte proliferation	In vitro	[60,62,63]

GM-CSF: Granulocyte-macrophage colony stimulating factors.

**Table 3:** Other functions of AR and its major constituents.

Parts	Biological Functions	Model	References
**Polysaccharides**	Anti-obesity	In vitro; In vivo	[72,73,74]
**Saponins**	Reduction of tumor size	In vivo	[75]
	Downregulation of mTOR expression and interference with DNA binding activity	In vitro	[76]
	Suppression of VEGF and bFGF levels and downregulation of p-Akt, p-mTaOR, VEGF, VEGFR1, and VEGFR2	In vitro; In vivo	[77]
	Enhancement of immune response	In vitro; In vivo	[78,79]
	Induction of BMP-2 and Smad1/5/8 expressions	In vitro	[80]

## Abbreviations

ABTS	2,2′-azino-bis(3-ethylbenzothiazoline-6-sulphonic acid
ALT	Alanine aminotransferase
AR	Astragali Radix
ASR	Angelica Sinensis Radix
AST	Aspartate aminotransferase
BYHWD	Buyang Huanwu decoction
CEC	Circulating endothelial cells
CMM	Chinese Materia Medica
DBT	Danggui Buxue Tang
DDP	Cisplatin
DMN	Dimethylnitrosamine
DPPH	2,2-Diphenyl-1-Picrylhydrazyl
EF	Epimedii Folium
EPO	Erythropoietin
ET-1	Endothelin-1
FRAP	Ferric reducing ability of plasma
GABA	γ-aminobutyric acid
HA	Hexadecenoic acid
HIF-1α	Hypoxia-induced factor
HNPCE	Homogenization-assisted negative pressure cavitation extraction
Hyp	Hydroxyproline
IDO	Indoleamine 2,3-dioxygenase
LN	Laminin
MAAH	Microwave-assisted acidic hydrolysis
MDA	Malondialdehyde
MMP	Matrix metalloproteinases
NSCLC	Non-small-cell lung cancer
PCIII	Procollagen type III
PLE	Pressurized liquid extraction
TCM	Traditional Chinese medicine
YPFS	Yu Ping Feng San
